# Direct measurement of intercellular CO_2_ concentration in a gas-exchange system resolves overestimation using the standard method

**DOI:** 10.1093/jxb/ery044

**Published:** 2018-02-08

**Authors:** Jun Tominaga, Hiroshi Shimada, Yoshinobu Kawamitsu

**Affiliations:** 1 Faculty of Agriculture, University of the Ryukyus, Okinawa, Japan; 2 Department of Mathematical and Life Sciences, Graduate School of Science, Hiroshima University, Japan

**Keywords:** *A–C*_i_ curve, amphistomatous, cuticle, *Heliunthus annuus*, hypostomatous, patchy stomatal closure, *Passiflora edulis*, *Phaseolus vulgaris*, photosynthesis, stomata

## Abstract

Intercellular CO_2_ concentration of leaves (*C*_i_) is a critical parameter in photosynthesis. Nevertheless, uncertainties in calculating *C*_i_ arise as stomata close. Here, by modifying the assimilation chamber of a commercial gas-exchange equipment to directly measure *C*_i_, we demonstrate overestimation of calculated *C*_i_ (i.e. *C*_i(c)_) without stimulating stomatal closure. Gas exchange was measured on one side of the leaf while measured *C*_i_ (*C*_i(m)_) was acquired simultaneously on the other side of the leaf in hypostomatous passion fruit (*Passiflora edulis* Sims) and amphistomatous sunflower (*Helianthus annuus* L.) and common bean (*Phaseolus vulgaris* L.). The adaxial surface showed comparable *C*_i(c)_ and *C*_i(m)_ in sunflower, whereas in common bean, where the adaxial surface has a low stomatal density, *C*_i(c)_ markedly differed from *C*_i(m)_ when the stomata remained open. However, the latter discrepancy disappeared when measuring the leaf flipped upside down so that the gas exchange was measured (i.e. *C*_i_ was calculated) on the abaxial side, which has a much higher stomatal density. The passion fruit showed the largest discrepancy on the astomatous side, indicating that the cuticle has a large impact on the calculation. Direct measurement of *C*_i_ is recommended as a more accurate estimate than the calculation when stomatal gas transport is restricted. Occurrence of overestimation and prospects for direct measurement are discussed.

## Teaser

Obtaining leaf intercellular CO_2_ concentration by direct measurement, which is affected by neither cuticle properties nor stomatal behavior, is better than by calculation, which largely neglects cuticular gas transport when stomatal gas transport is restricted

## Introduction

In land plants, leaves constitute the major interface with the atmosphere. Inside leaves, mesophyll cells consume CO_2_ during photosynthetic assimilation (*A*), and consequently the CO_2_ concentration in the intercellular airspace (*C*_i_) is lower than in the bulk air outside the leaf (*C*_a_). The CO_2_ enters leaves by diffusing through stomatal pores on the leaf surface, so *C*_i_ essentially indicates the CO_2_ substrate available for *A* (Farquhar *et al.*, 1980). The cells inside leaves are wet, and the stomata allow water vapor to escape by transpiration. Although stomata can close if dehydration becomes excessive, CO_2_ entry is also restricted by stomatal closure, thereby diminishing *A*. Therefore, the response of *A* to various *C*_i_ (i.e. the *A*–*C*_i_ curve) is the foundation for relating photosynthetic biochemistry to prevailing environmental conditions experienced by leaves (Hanson *et al.*, 2016).

In general gas-exchange measurements, *C*_i_ is calculated from the relationship between water vapor exiting and CO_2_ entering through stomata (Moss and Rawlins, 1963). Instead, Sharkey *et al.* (1982) measured *C*_i_ directly. They put amphistomatous leaves between a chamber and a cup and measured the CO_2_ concentration inside the cup after it had equilibrated. Because no net CO_2_ exchange occurred through that side of the leaf, the CO_2_ concentration measured in the cup would be close to the concentration inside the leaf (measured *C*_i_; *C*_i(m)_). Indeed, the *C*_i(m)_ was identical to the value calculated (calculated *C*_i_; *C*_i(c)_) on the other side.

Subsequently, close comparisons of *C*_i(m)_ and *C*_i(c)_ have highlighted the gradient of intercellular CO_2_ in the airspace owing to the finite conductance of the airspace (Mott and O’Leary,1984; Parkhurst *et al.*, 1988). The gradient varies between species, depending on the anatomy of the mesophyll pores (Evans *et al.*, 2009). For species having a fast assimilation rate, such as sunflower (*Helianthus annuus* L.), the gradient is generally too small to affect *C*_i(m)_ (Mott and O’Leary, 1984; Boyer and Kawamitsu, 2011). The small effect observed for sunflower leaves was confirmed recently when a direct-measurement system for *C*_i_ (Boyer and Kawamitsu, 2011) was incorporated into a commercial open gas-exchange apparatus (Tominaga and Kawamitsu, 2015*a*). Using a similar system, however, Tominaga and Kawamitsu (2015*b*) demonstrated that *C*_i(c)_ markedly differed from *C*_i(m)_ as stomata began to close after abscisic acid (ABA) was applied to this species. Tracing *A*–*C*_i_ curves, the *C*_i(c)_ showed an artefactual limitation of photosynthesis, as was previously indicated for ABA-fed leaves (Downton *et al.*, 1988; Terashima *et al.*, 1988; Meyer and Genty, 1998), whereas the *C*_i(m)_ showed a similar curve to leaves with open stomata. Therefore, these investigators attributed this difference to overestimation of the calculation.

For the calculation, the diffusivity ratio of trace gases (water vapor, CO_2_) through the leaf surface is considered constant—assuming stomata are the dominant path for both gasses (Moss and Rawlins, 1963, von Caemmerer and Farquhar, 1981; Boyer and Kawamitsu, 2011). Using hypostomatous leaves of grape (*Vitis vinifera* L.), however, small gas fluxes on the astomatous adaxial side (i.e. cuticle/epidermis) were detected while the opposite stomatous surface was sealed (Boyer *et al.*, 1997; Boyer, 2015*a*). At the same time, *C*_i(c)_ was much larger than expected (the photosynthetic CO_2_ compensation point). Evidently, this discrepancy was a consequence of the cuticle, which transmits water vapor 20- to 40-fold faster than it transmits CO_2_, a difference that is considerably larger than the constant 1.6 for stomata. Measuring *C*_i_ directly in sunflower, Boyer (2015*b*) calculated cuticular transpiration on the stomatous surface and suggested that it could significantly overestimate CO_2_ entry. Likewise, Tominaga and Kawamitsu (2015*b*) calculated the cuticular conductance of water vapor and reached the same conclusion.

On the other hand, a number of studies have attributed the overestimation of *C*_i_ to a heterogeneous stomatal aperture or patchy stomatal closure. This likely occurs in response to acute stimuli (Gunasekera and Berkowitz, 1992), including application of ABA (Downton *et al.*, 1988; Terashima *et al.*, 1988), casting doubt upon the conclusion made about stomatous leaf surfaces. In fact, it is difficult to determine whether a suspicious value for *C*_i_ can be attributed to cuticular transport or to stomatal patchiness because both effects may appear simultaneously as stomata close. Another factor of concern is the intercellular CO_2_ gradient because any direct measurement inevitably limits CO_2_ entry from one side, thereby increasing the gradient. Finally, in evaluations of the cuticle effect on the calculation (Boyer, 2015*b*; Tominaga and Kawamitsu, 2015*b*), CO_2_ transport through the cuticle was assumed to be negligible; however, this might be an oversimplification.

We therefore conducted experiments to investigate the effect of the cuticle on the calculation without stimulating stomatal closure. Gas exchange was measured separately on the adaxial and abaxial leaf surfaces of common bean (*Phaseolus vulgaris* L.), for which the stomatal density differs on each side. Simultaneously, *C*_i(m)_ was acquired for the other side. For comparison with common bean, we measured symmetric amphistomatous leaves of sunflower (*Heliunthus annuus* L.) and hypostomatous leaves of passion fruit (*Passiflora edulis* Sims).

## Materials and methods

### Plant material

Common bean (*P. vulgaris* L. cv. Kentucky Blue) and sunflower (*H. annuus* L. cv. Hybrid sunflower) plants, each 5–6 weeks old, were sources of amphistomatous leaves, and 1-year-old purple passion fruit (*P. edulis* Sims) plants were the source of hypostomatous leaves. Passion fruit plants were grown in 12-liter plastic pots, and the bean and sunflower plants were grown in 4-liter plastic pots; each pot contained a soil mixture (soil:peat:sand=1:1:1) in a greenhouse located at the University of the Ryukyus, Okinawa, Japan (26°15′N, 127°45′E; elevation 127 m). The growth irradiance depended on solar radiation, and the daily maximum ranged from 300 to 1500 μmol m^–2^ s^–1^ photosynthetically active radiation (PAR) for sunflower and common bean. Passion fruit was grown under a shade net and received approximately 30% less irradiance than the other species. The daily maximum and minimum growth temperatures were, on average, 30.9 °C and 16.2 °C, respectively. Water was supplied whenever the soil surface was dry. All soil was saturated weekly with Hoagland’s nutrient solution composed of 4 mM KNO_3_, 6 mM Ca(NO_3_)_2_·4H_2_O, 2 mM MgSO_4_·7H_2_O, 2 mM KH_2_PO_4_, 0.5 µM CuSO_4_·5H_2_O, 10 µM MnSO_4_·H_2_O, 2 µM ZnSO_4_·7H_2_O, 25 µM H_3_BO_3_, 0.5 µM H_2_MoO_4_ and 0.5 µM Fe(III)-EDTA. All experiments with these plants were conducted with fully expanded leaves.

### Gas-exchange and direct-measurement systems

**Fig. 1.**
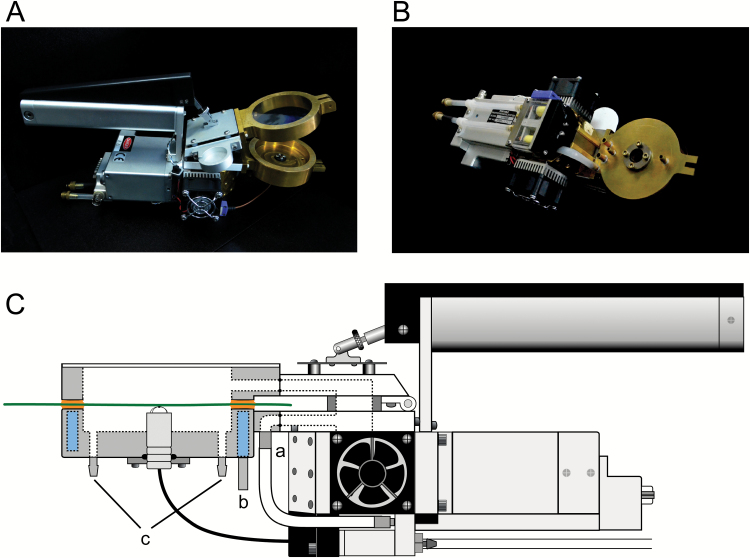
The lab-made chamber/cup for direct measurement of intercellular CO_2_ concentration (*C*_i_). View from (A) front side and (B) back side, and (C) schematic diagram of the chamber/cup clamped on a leaf (represented by the green horizontal line). The brass chamber/cup was specially designed for the sensor head of the Li-Cor LI-6400XT open gas-exchange system. The leaf separates the cup from the chamber. The leaf-clamp seal is ensured by a coating of paraffin/lanolin. Mixing air passes through the upper chamber, allowing the leaf surface to exchange gases that are exhausted through the bypass (a). Water circulated inside the chamber wall (b) controls air temperature in the cup. A small blower circulates the air in the cup to the gas analyser (LI-840A; Li-Cor) through the inlet and outlet located at the bottom of the cup (c). The round chamber window (28.3 cm^2^) is covered with Propafilm (250-01885, Li-Cor). A leaf-temperature thermocouple (6400-04; Li-Cor) is inserted into the cup and attached to the lower surface of the leaf, as is the case in the standard LI-6400 chamber.

The gas-exchange system has been reported in detail elsewhere (Tominaga and Kawamitsu, 2015*a*), and we used a modified system (the design drawings are available upon request). Briefly, the small cup of the Tominaga and Kawamitsu system was replaced with the bottom half of an assimilation chamber (LI6400-40; Li-Cor, Lincoln, NE, USA) of a commercial gas-exchange system (LI-6400XT; Li-Cor). Although use of the commercial assimilation chamber has certain advantages (e.g. combined fluorescence measurement), the small chamber is especially prone to diffusion leaks (Rodeghiero *et al.*, 2007). To minimize the effect of leakage and detect small fluxes accurately, we used a larger chamber/cup that could also be attached to the sensor head (Fig. 1). This new laboratory-made chamber/cup with a window diameter of 6 cm encloses a 14-fold larger leaf area (28.3 cm^2^) than the LI-6400-40 chamber (2 cm^2^) with the window diameter of 0.8 cm. The chamber window is covered with Propafilm (polypropylene that is coated with Saran; 250-01885, Li-Cor).

The new chamber/cup was replaced with the upper or lower half of the chamber (Fig. 1). A paraffin/lanolin mixture (2:8) was spread evenly (thickness ~1 mm) on the rims of both the chamber and the cup (i.e. the interface). This semi-solid coat sealed the chamber tightly when a leaf was clamped. All experimental leaves were large enough to cover the entire window area so that the upper chamber and lower cup were divided by the leaf. In the cup, CO_2_ equilibrated with that inside the stomatal pores (*C*_i(m)_). The equilibrated air was smoothly circulated with a small fan to the infrared gas analyser (IRGA; LI-840A; Li-Cor) in a closed loop. To compensate for the volume of the larger cup, which potentially could delay the equilibration, the path length of the loop was kept as short as possible (~40 cm). The overall volume of the closed loop for the new system was 137 ml (1.2-fold larger than the previous system) determined by monitoring the concentration change with the LI-840A after injecting a small amount of 5% CO_2_ gas into the loop (see Supplementary Fig. S1 at *JXB* online). In the loop, *C*_i(m)_ was continuously measured while gas exchange through the opposite side of same leaf area was detected with the open gas-exchange system, which automatically calculated *C*_i_ (see ‘Calculations’). Accordingly, both *C*_i(m)_ and *C*_i(c)_ were obtained simultaneously for the same leaf area, allowing close comparison. Using a water-saturated filter paper (Advantec No. 1) as a leaf model (Ball, 1987), the boundary layer conductance for the lab-made chamber was estimated to be 1.38 mol m^–2^ s^–1^, which was less than one-third that of the LI-6400-40 and yet sufficiently greater than the stomatal conductance.

### Gas-exchange measurements

After reaching steady state photosynthesis at the ambient CO_2_ concentration of 300–400 μmol per mole of air (hereafter μmol mol^–1^), the concentration was changed stepwise, allowing *C*_i_ to vary. CO_2_ concentration was regulated by mixing pure CO_2_ supplied to the LI-6400 console with CO_2_-free air humidified with a dew-point generator (LI-610; Li-Cor). Each leaf was illuminated with three metal-halide lamps (D-400, Toshiba) located approximately 1 m above the chamber. Fine net shades were inserted between the light source and the chamber so that the irradiance was adjusted to 300 μmol m^–2^ s^–1^ PAR, which was less than the 800 μmol m^–2^ s^–1^ PAR used in a previous study (Tominaga and Kawamitsu, 2015*a*,b). We reduced the irradiance to control leaf temperature as the light source containing a broad thermal ray otherwise warmed the leaf. As a result of the lower irradiance, the *A* for sunflower leaves was below saturation and yielded a lower *A*/*C*_i_ ratio of 0.064 (*C*_i_=220 μmol mol^–1^) than the value of 0.09 (*C*_i_=280 μmol mol^–1^) measured previously (Tominaga and Kawamitsu, 2015*b*). All gas-exchange measurements were carried out at a leaf temperature of 25 ± 0.1 °C, a leaf to air vapor pressure deficit of <1.5 kPa, and a constant gas flow rate of 500 μmol s^–1^. Common bean and sunflower leaves responded by slowly closing their stomata when the CO_2_ concentration was somewhat higher than the ambient level, which often required a longer measurement time. To help decrease the measurement time, the ambient CO_2_ concentration was increased to no more than 700 μmol mol^–1^.

### Diffusion leaks

The upper chamber and lower cup were separated by clamping aluminum foil (instead of the leaf in Fig. 1C) using the paraffin/lanolin coat. For the open gas-exchange system, a test for diffusion leaks was carried out by estimating CO_2_ and the water vapor diffusion molar flow rate, *K*_CO2_ and *K*_H2O_, respectively, according to Rodeghiero *et al.* (2007). To maintain the concentration gradients of CO_2_ and water vapor between the outside and inside of the chamber, the sensor head was enclosed by a semi-closed cylinder in which air containing 400 μmol mol^–1^ CO_2_ and 20 mmol mol^–1^ water vapor (monitored with the LI-840A) flowed continuously at a rate of 15 l min^–1^ whereas dry air with 30 μmol mol^–1^ CO_2_ was supplied to the chamber from the console. *K*_CO2_ and *K*_H2O_

were estimated as 0.21 ± 0.079 and 0.71 ± 0.13 μmol s^–1^, respectively, values that were comparable to those determined for the smaller LI-6400-40 chamber (Rodeghiero *et al.*, 2007; Tominaga and Kawamitsu, 2015*a*), suggesting a similar rate of diffusion leak on a chamber basis. Accounting for the larger chamber interface (i.e. circumference of the chamber window), the new chamber had a better seal. Because the leakage can cause gas fluxes that can affect both assimilation and transpiration, which are measured on a leaf-area basis, the apparent fluxes (leak rate divided by leaf area) were significantly decreased in the larger chamber as compared with the previous system. The CO_2_ and water vapor concentrations outside the chamber were monitored by open-path IRGA (LI-7500; Li-Cor) set around the leaves, and were subsequently used to correct for leaks as described in Rodeghiero *et al.* (2007). The apparent fluxes in the open system were estimated to be <0.03 μmol m^–2^ s^–1^ for CO_2_ and <0.001 mmol m^–2^ s^–1^ for water vapor throughout the measurements. In most of our experiments, such small fluxes changed the calculation of *C*_i_ by only <1%.

The CO_2_ leakage in the closed system increases or decreases the equilibrium of *C*_i(m)_ depending on the concentration gradient relative to the outside. To detect the leakage, we monitored the change of CO_2_ concentration in the empty cup (*C*_cup_) after partially enclosing the expired air containing a high concentration of CO_2_ of ~1800 μmol mol^–1^ (see Supplementary Fig. S2). The leak was indicated by the *C*_cup_ decreasing slowly at a rate of 10.8 μmol mol^–1^ h^–1^ while the outside CO_2_ concentration (*C*_room_) was constant at ~400 μmol mol^–1^. Based on this leak rate and the known volume of the closed system (137 ml), the rate of CO_2_ diffusion leak was estimated to be 0.0054 μmol m^–2^ s^–1^ on a leaf-area basis and should be even lower when *C*_cup_ is closer to *C*_room_ (*c.* 400–500 μmol mol^–1^) in our experiments. The CO_2_ leakage occurred too slowly to affect the equilibrium, and thus no correction was applied to the *C*_i(m)_.

### Stomatal density and size

After gas-exchange measurements, nail polish was thinly spread at several points on both the upper and lower leaf surfaces within the measured leaf area. After waiting for the polish to dry, the polish was striped with clear double-sided tape and attached to a glass slide (i.e. a replica of the epidermis). Microphotographs of the replica were taken with a digital camera (HDR-SR12; Sony, Tokyo, Japan) attached to a microscope (Eclipse 80i; Nikon, Kawasaki, Japan). For each side of the leaves, 20 microphotographs were taken at random. We counted the number of stomata in each microphotograph (0.64–1.82 mm^2^) to calculate stomatal density. Mean stomatal size was calculated based on the length of the long axis of the stomata.

### Calculations

Realizing that departing vapor behaved like entering CO_2_, Moss and Rawlins (1963) first calculated *C*_i_ (*C*_i(c)_) from measured variables:

$$C_{\text{i(c)}}=C_{\text{a}}-1.6\frac{A}{E}(w_{\text{i}}-w_{\text{a}})$$ (1)

where *C*_a_ is the CO_2_ concentration in the air (mol mol^–1^), *A* is the assimilation rate for CO_2_ moving into the leaf through stomata (mol m^–2^ s^–1^), *E* is the transpiration rate for water vapor moving out of the leaf through stomata (mol m^–2^ s^–1^), and *w*_i_ and *w*_a_ are the water vapor concentrations in the leaf and air outside the leaf, respectively (mol mol^–1^). The only other factor needed was the ratio of the diffusivity of water vapor to that of CO_2_, which is ~1.6 in air (Jarvis, 1971; Massman, 1998) assuming that stomata are the dominant path for both gases.

Von Caemmerer and Farquhar (1981) rigorously extended the Moss–Rawlins relation to account for interactions between water vapor and CO_2_:

$$C_{\text{i(c)}}=C_{\text{a}}-1.6\frac{A+\bar{C}E}{E-\bar{w}E}(w_{\text{i}}-w_{\text{a}})$$ (2)

where $\bar{C}E$and $\bar{w}E$($\bar{C}$=(*C*_a_ + *C*_i(c)_)/2 and $\bar{w}$=(*w*_a_ + *w*_i_)/2) are interaction terms. This equation became the norm for determining *C*_i_ and is routinely used in commercial gas-exchange units including the LI-6400. Note that both *C*_a_ and *w*_a_ were implicitly located in the boundary layer of the leaf surface in this study. The *C*_a_ and *w*_a_ were, respectively, estimated with the boundary layer conductance of water vapor (1.38 mol m^–2^ s^–1^) and that of CO_2_ (1.01 mol m^–2^ s^–1^) estimated from the ratio of diffusivity in the ‘convective air’ (1.37) rather than ‘still air’ (1.6) for the stomatal pore (Ball, 1987). Von Caemmerer and Farquhar (1981) expressed the diffusion property of water vapor in Eq. (2) as a form of conductance (*g*_w_):

$$g_{\text{w}}=\frac{E-\bar{w}E}{w_{\text{i}}-w_{\text{a}}}$$ (3)

In contrast to Eq. (2), the *C*_i(m)_ already reflected the interactions of CO_2_ and water vapor (Boyer and Kawamitsu, 2011):

$$C_{\text{i(m)}}=C_{\text{a}}-1.6\frac{A}{E-\bar{w}E}(w_{\text{i}}-w_{\text{a}})$$ (4)

where the only difference from Eq. (2) is the absence of the term $\bar{C}E$. Boyer and Kawamitsu (2011) first expressed conductance of CO_2_ (*g*_c_) in terms of directly measured variables:

$$g_{\text{c}}=\frac{A}{C_{\text{a}}-C_{\text{i(m)}}}$$ (5)

In this study, the conductance of water vapor (*g*_w_) and of CO_2_ (*g*_c_) was derived from the directly measured variables using Eqs (3) and (5), respectively.

## Results

### Stomatal density and size

In leaves of the common bean, the adaxial side had significantly fewer stomata (9 ± 5 mm^–2^) than the abaxial side (274 ± 31 mm^–2^), whereas in sunflower the stomata were distributed more evenly on both sides (Table 1). On the adaxial side, the stomatal density in sunflower (221 ± 24 mm^–2^) is much higher than in common bean. The stomatal ratio (adaxial/abaxial) was 0.03 for common bean leaves and 0.85 for sunflower. Stomatal size did not differ significantly between the two sides in sunflower, whereas in the common bean the adaxial surface had 33% larger stomata than the abaxial surface. Passion fruit leaves, which were observed to bear stomata only on the abaxial surface, had a stomatal density (209 ± 26 mm^–2^) comparable to that of sunflower.

**Table 1.** Stomatal density and size in passion fruit, sunflower, and common bean

	Adaxial	Abaxial	*P*	Ratio (adaxial/abaxial)
Stomatal density (mm^–2^)				
Passion fruit	0 (*n=*80)	209 ± 26 (*n*=80)		
Sunflower	188 ± 25 (*n*=80)	221 ± 24 (*n*=80)	<0.001	0.85
Common bean	9 ± 5 (*n*=160)	274 ± 31 (*n*=160)	<0.001	0.032
Long axis of stomata (μm)				
Passion fruit		10.7 ± 1.3 (*n*=40)		
Sunflower	12.4 ± 1.8 (*n*=40)	12.2 ± 2.7 (*n*=40)	0.583	1.02
Common bean	10.9 ± 1.7 (*n*=80)	8.2 ± 1.3 (*n*=80)	<0.001	1.33

The values are the mean ±SD.

### Hypostomatous leaves

**Fig. 2.**
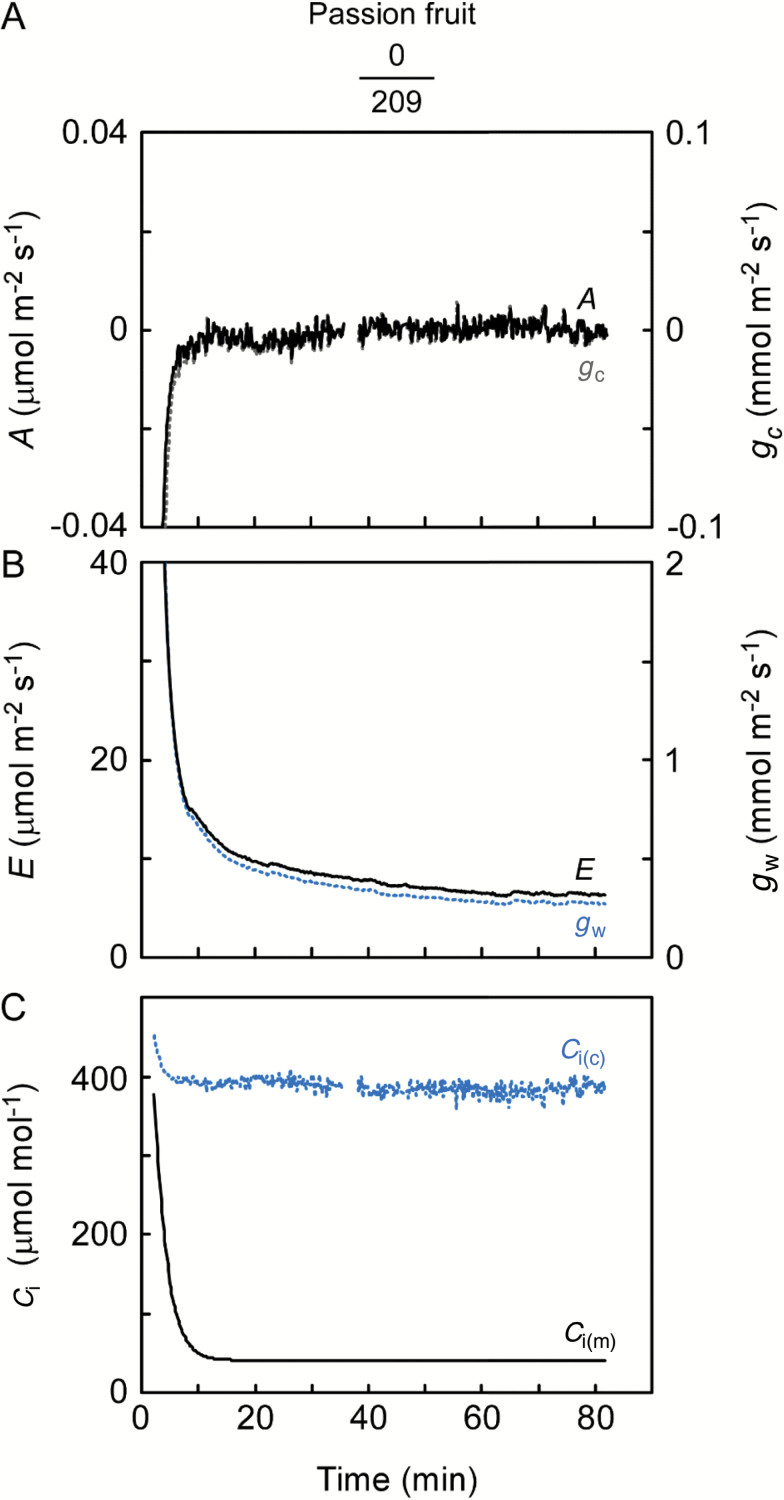
Change in (A) assimilation rate (*A*) and conductance of CO_2_ (*g*_c_), (B) transpiration rate (*E*) and conductance of water vapor (*g*_w_), and (C) intercellular CO_2_ concentration (*C*_i_) after clamping a passion fruit leaf. Numbers below species name indicate stomatal densities (Table 2) for the leaf side attached to the assimilation chamber (upper number) and the cup (lower number); gas exchange through the astomatous side was measured in the chamber while *C*_i_ was measured directly (*C*_i(m)_) in the cup. Calculated *C*_i_ (*C*_i(c)_) was simultaneously derived from the transpiration occurring within the same leaf area of the other adaxial surface. Note that *A* and *g*_c_ overlap one another. In (A) and (C), erroneous data that were attributable to CO_2_ control by LI-6400 were removed. The experiments were conducted at a constant leaf temperature of 25 °C and ambient CO_2_ concentration of 400 μmol mol^–1^. Representative experiment from four replications.

**Fig. 3.**
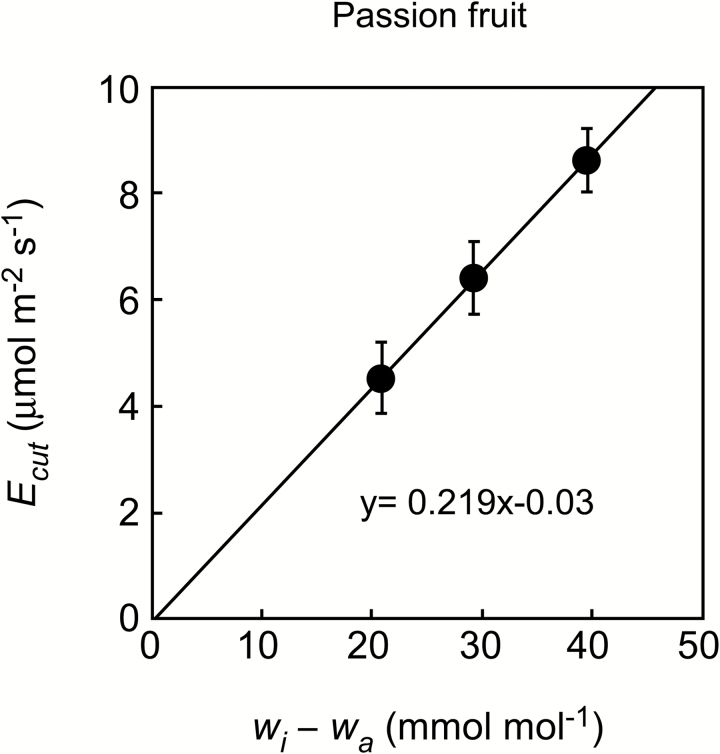
Relationships between cuticular transpiration (*E*_cut_) and gradient of water vapor concentration between the inside (*w*_i_) and outside (*w*_a_) of a passion fruit leaf. *E*_cut_ was measured on the adaxial astomatous surface. The water vapor gradient varied with leaf temperature (25, 29, 33 °C) when *w*_a_ was kept constant. Means ±SE (*n*=4) and a regression line are shown.

We placed the cup on the abaxial stomatous side while photosynthesis and transpiration were measured simultaneously in the chamber on the adaxial astomatous side of passion fruit leaves. Soon after clamping the leaf, both assimilation rate (*A*) and conductance of CO_2_ (*g*_c_) became too small to be detected on the astomatous side (Fig. 2A). In contrast, both transpiration rate (*E*) and conductance of water vapor (*g*_w_) were detectably large on the same side (Fig. 2B). These results demonstrated that the cuticle loses more water vapor than the amount of entering CO_2_. On the stomatous side, photosynthesis diminished the amount of CO_2_ inside the cup until equilibrium was reached (Fig. 2C). Because CO_2_ scarcely entered from the astomatous surface (Fig. 2A), the CO_2_ concentration at equilibrium (*C*_i(m)_) ought to be equivalent to the photosynthetic CO_2_ compensation point where no net flux occurs in the leaf. In agreement with this supposition, *C*_i(m)_ equilibrated to ~43 μmol mol^–1^ at a leaf temperature (*T*_leaf_) of 25 °C. In contrast, *C*_i_ calculated from the transpiration (*C*_i(c)_) remained high, differing substantially from *C*_i(m)_. *E* on the astomatous surface denotes cuticular transpiration (*E*_cut_), and on average *E*_cut_ was 4.25 μmol m^–2^ s^–1^ at a *T*_leaf_ of 25 °C. While keeping the ambient vapor concentration (*w*_a_) constant, the vapor concentration inside the leaf (*w*_i_) was raised stepwise by heating the leaf to 29 °C and subsequently to 33 °C (see Supplementary Fig. S3). The difference of the vapor concentration gradients across the cuticle (*w*_i_–*w*_a_) increased from 21 mmol mol^–1^ (at *T*_leaf_ of 25 °C) to 29 and 39 mmol mol^–1^, respectively. *E*_cut_ correlated positively and linearly with the vapor concentration gradients (Fig. 3). We calculated the cuticular conductance of water vapor ($g_{\text{w}}^{\text{cut}}$) on the astomatous surface according to $g_{\text{w}}^{\text{cut}}$=*E*_cut_/(*w*_i_ – *w*_a_) (Boyer *et al.* 1997). The $g_{\text{w}}^{\text{cut}}$ was 0.21 mmol m^–2^ s^–1^ at each leaf temperature (Table 2) and also coincided well with the slope of the regression in Fig. 3. The constant $g_{\text{w}}^{\text{cut}}$ indicated that the cuticle was stable at physiological temperatures (25–33 °C), supporting the previous study of isolated cuticles (Riederer and Schreiber, 2001). On the stomatous side, *C*_i(m)_ closely followed leaf temperature (Supplementary Fig. S3). The value and temperature dependence of *C*_i(m)_ (Table 2) were similar to those of the intercellular CO_2_ photocompensation point (*C*_i_*) for *C*_3_ species (Walker and Ort, 2015). In this experiment, the smaller *w*_a_ compared with *w*_room_ potentially caused leakage of water vapor into the chamber. The effect on *E*_cut_ was estimated to be greatest (7.6–13.3% of the apparent flux) when *E*_cut_ was small at a *T*_leaf_ of 25 °C and reached a minimum (4.6–6.0%) when *E*_cut_ was high at a *T*_leaf_ of 33 °C.

**Table 2.** Temperature responses of $g_{\text{w}}^{\text{cut}}$, *C*_i(m)_, and *C*_i(c)_

*T*_leaf_ (°C)	$g_{\text{w}}^{\text{cut}}$ (μmol m^–2^ s^–1^)	*C*_i(m)_ (μmol mol^–1^)	*C*_i(c)_ (μmol mol^–1^)
25	213 ± 33	42.7 ± 0.4	469 ± 12
29	214 ± 23	51.4 ± 0.4	391 ± 10
33	211 ± 14	62.1 ± 1.0	330 ± 3

The values are the mean ±SE (*n*=4).

### Amphistomatous leaves

**Fig. 4.**
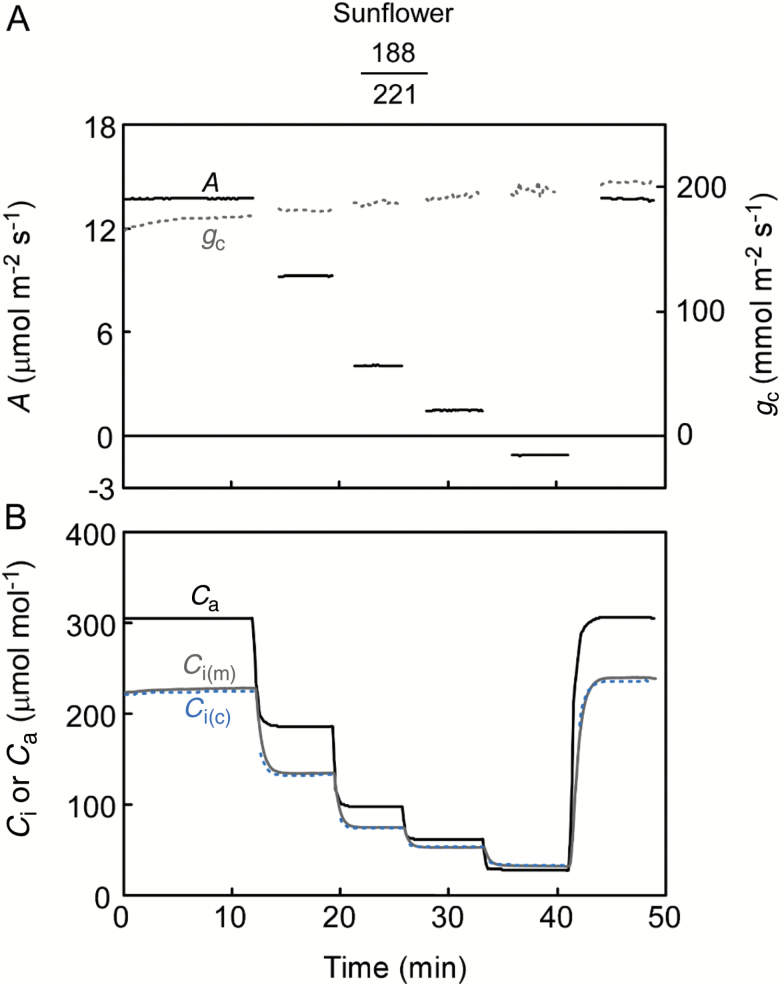
Response of (A) assimilation rate (*A*) and conductance of CO_2_ (*g*_c_) to (B) various *C*_i_ for sunflower leaves. Gas exchange through the adaxial side was measured. The change in ambient CO_2_ concentration (*C*_a_) is also shown with *C*_i_. Values for *C*_i(m)_ and *C*_i(c)_ overlapped one another. For the gas-exchange measurement (A), the data acquired immediately after the change in *C*_a_ were removed owing to extreme values. Representative experiment from four replications.

**Fig. 5.**
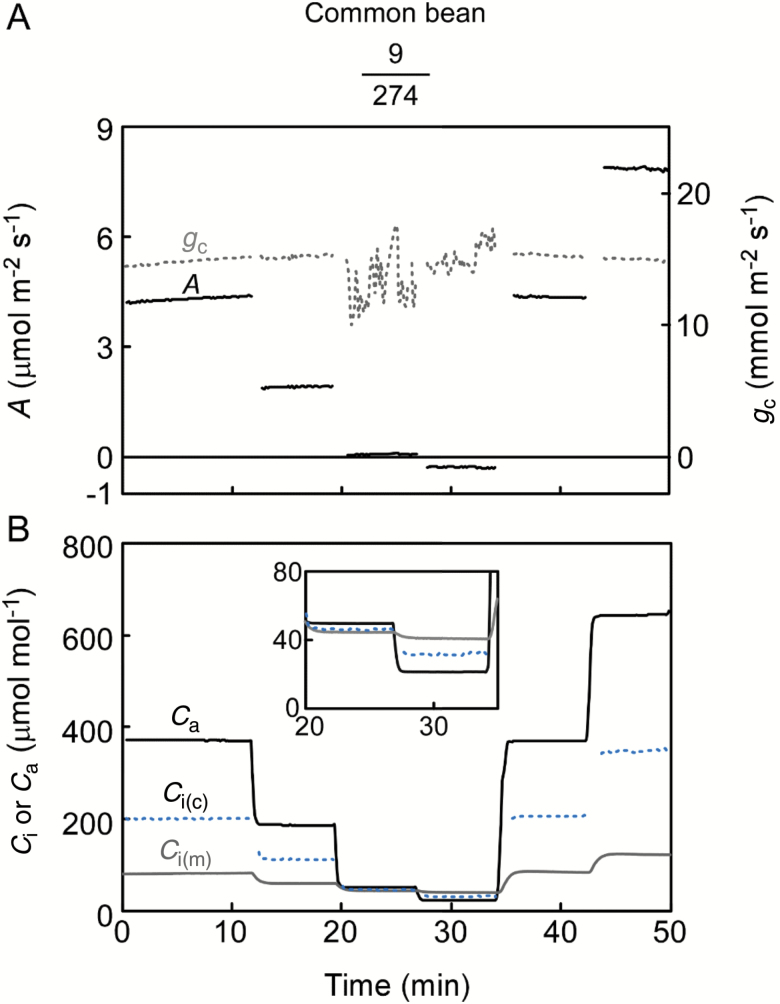
As in Fig. 4, *A*–*C*_i_ curve measurement for common bean leaves. Gas exchange through the adaxial side was measured. Representative experiment from four replications.

**Fig. 6.**
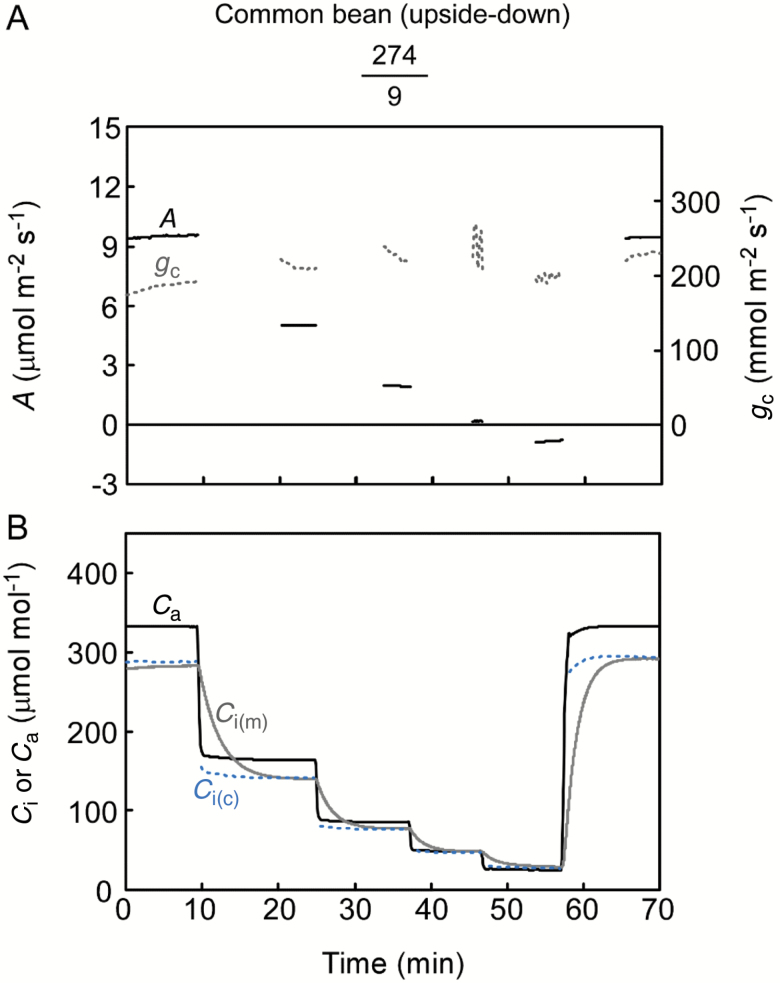
*A*–*C*_i_ curve measurement for common bean leaves. Each leaf was flipped upside-down so that the gas exchange through the abaxial side could be measured. Representative experiment from four replications.

The adaxial side of leaves was set up to allow gas exchange, and we measured *A*–*C*_i_ curves in sunflower—the species previously assessed with a smaller chamber and cup (Tominaga and Kawamitsu, 2015*a*,b). The adaxial stomata remained open during the experiments as indicated by the relatively constant *g*_c_ (Fig. 4A). *A* was rapidly balanced with *C*_i(c)_ at each *C*_a_. As expected for open stomata (*g*_c_=178 ± 24 mmol m^–2^ s^–1^), the calculation coincided well with the direct measurement for this species (Fig. 4B). It was clear that the larger cup facilitated a response of *C*_i(m)_ as fast as *C*_i(c)_. We then carried out the experiment using common bean leaves, for which the stomatal ratio contrasts with that of sunflower leaves (Table 1). With few stomata on the adaxial surface, *g*_c_ was <20 mmol m^–2^ s^–1^ (Fig. 5A). A sensitive response of *A* to CO_2_ indicates open stomata. Comparison with *g*_w_ suggested that *g*_c_ became noisy when *C*_a_ was small (see Supplementary Fig. S4) due to the detection limit of *A* but not the *C*_i(m)_ (see Discussion). The gradient between *C*_a_ and *C*_i(m)_ appropriately directed the subtle CO_2_ flux, demonstrating the accuracy of the measurement, e.g. *C*_i(m)_ is always lower than the *C*_a_ when *A* is positive and vice versa. The *C*_i(c)_ differed largely from the *C*_i(m)_ from the outset (Fig. 5B). The effect of leak corrections on the *C*_i(c)_ was <4%. To further investigate the cause of this discrepancy, we flipped the leaf so that CO_2_ could readily diffuse into the abaxial side having much greater stomatal density (Fig. 6). The *g*_c_ was 222 ± 15 mmol m^–2^ s^–1^, and the average CO_2_ conductance ratio (abaxial/adaxial) was 0.073. This value was more than double that of the stomatal ratio (0.032), suggesting that the stomata on the adaxial surface, although fewer in number, opened wider perhaps because of their larger size (Table 1). It took longer for *C*_i(m)_ to equilibrate for the flipped leaves because the CO_2_ in the cup diffused more slowly through the adaxial side with few stomata (cf. Figs 5 and 6). At equilibrium, the *C*_i(m)_ and *C*_i(c)_ were similar (Fig. 6B).

### Diffusivity ratio of water vapor and CO_2_ of the leaf surface

**Fig. 7.**
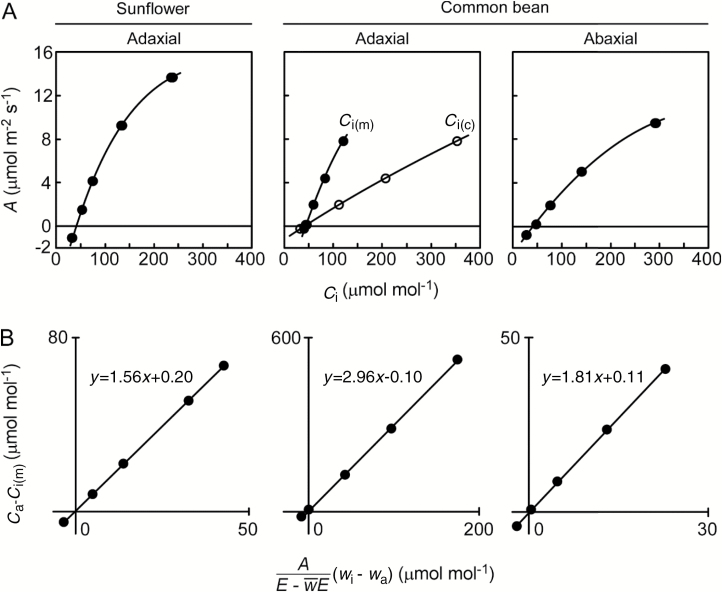
Data from Figs 2–4 using sunflower and common bean plotted as (A) *A*–*C*_i_ curves and (B) relationships derived from the measured variables in Eq. (6). In (A), closed circles denote *C*_i(m)_ and open circles denote *C*_i(c)_. Note that *C*_i(m)_ and *C*_i(c)_ are on top of each other for adaxial leaf side of sunflower and abaxial leaf side of common bean. The slope in (B) indicates the diffusivity ratio through the leaf surface (water/CO_2_).

After plotting *A* against *C*_i(m)_ and *C*_i(c)_ for the experiments described above, suppressed *A*–*C*_i_ curves were then derived from *C*_i(c)_ for the common bean leaves, for which gas exchange was measured on the adaxial side (Fig. 7A). The similar result for this species was also observed previously using the small chamber/cup system (see Supplementary Fig. S5). Evidently, the large discrepancy between the *C*_i(c)_ and *C*_i(m)_ was not induced by patchy stomatal closure because the adaxial stomata remained open (Fig. 5). Assuming that this discrepancy is created by the intercellular CO_2_ gradients (i.e. *C*_i(c)_–*C*_i(m)_), flipping the leaf should impose the gradient to a similar degree to achieve the same *A* while keeping the intercellular conductance unchanged. In fact, this was not true (abaxial in Fig. 7A), and even comparable *C*_i(c)_ and *C*_i(m)_ suggested minute gradients. Consequently, the calculation overestimated *C*_i_ by neglecting cuticular gas transport since other possibilities were likely ruled out.

Upon measurement of leaf gas exchange, calculations essentially rely on the diffusivity ratio (water vapor/CO_2_) for stomata [1.6 in Eqs (1) and (2)], assuming that stomata are the only path for both water vapor and CO_2_. To measure the impact of the cuticle on the calculations, the diffusivity ratio through the leaf surface (*D*_w/c_) was estimated from directly measured variables based on Eq. (4):

$$C_{\text{a}}-C_{\text{i(m)}}=D_{\text{w/c}}\frac{A}{E-\bar{w}E}(w_{\text{i}}-w_{\text{a}})$$ (6)

where *D*_w/c_, instead of a theoretical constant 1.6 in Eq. (4), indicates the slope of the regression line for this relationship in Fig. 7B. For sunflower leaves, *D*_w/c_ was 1.58 ± 0.05. For common bean, values of *D*_w/c_ were 2.44 ± 0.37 and 1.79 ± 0.12 for the adaxial and abaxial side, respectively.

## Discussion

Our results demonstrate that the cuticle is an essential contributor to the large discrepancy between *C*_i(c)_ and *C*_i(m)_, supporting the conclusion reached with different species using different approaches (Boyer, 2015*b*; Tominaga and Kawamitsu, 2015*b*). In those previous experiments, ABA was fed to sunflower leaves to close stomata, thereby simulating the astomatous leaf surface. Although the stomatal closure is imperfect, the calculation overestimates *C*_i_ whereas the direct measurement is unaffected by the open/closed status of stomata. Here, we employed common bean leaves with few stomata on one side, i.e. instead of manually inducing stomatal closure, and we also observed a large discrepancy (Figs 5 and 7A), as seen with stomatal closure in sunflower leaves (Boyer, 2015*b*; Tominaga and Kawamitsu, 2015*b*). These stomata remained open even wider than those on the opposite, more stomatous surface, rejecting the possibility of patchy stomatal closure. Moreover, the discrepancy was not a consequence of the intercellular CO_2_ gradient because it disappeared for the flipped leaf with the intercellular conductance unchanged. These results were consistent with the evidence for passion fruit that clearly shows that cuticular gas transport is not negligible (Fig. 2).

### Implications of the direct measurements

Instead of diffusing through pores, at the outer cuticle layer both CO_2_ and water vapor diffuse through solid wax, which creates most of the resistance to cuticular gas transport (Šantrůček *et al.*, 2004). Consequently, the cuticle has a much smaller conductance than stomata despite covering most of the leaf surface (Table 2), and therefore the impact on the calculation is not substantial unless the stomatal gas transport diminishes in stomatous surfaces.

The cuticle effect appears because cuticle transports more water vapor than CO_2_ (Boyer *et al.*, 1997; Boyer, 2015*a*), as can be measured in the astomatous gas exchange (Fig. 2). The effect was still detectable for the stomatous leaf surface having *D*_w/c_ larger than 1.6 (Fig. 7B). Those *D*_w/c_ values were much less than those of 20–40 for the astomatous side of grape leaves (Boyer *et al.*, 1997; Boyer, 2015*a*) because the stomata still accounted for gas exchange to some extent. Similarly, *D*_w/c_ was 5.5 when the cuticular transpiration was estimated to be 5–16% of the total transpiration for sunflower leaves fed with ABA (Boyer, 2015*b*).

**Fig. 8.**
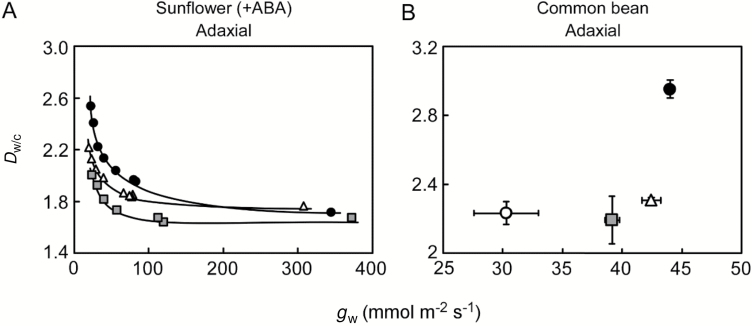
Relationships between conductance of water vapor (*g*_w_) and diffusivity ratio through the leaf surface (*D*_w/c_) estimated in *A*–*C*_i_ curve measurements for adaxial side of (A) sunflower leaves fed with 10 μM ABA and (B) common bean leaves. Different symbols represent replications with different leaves. Data shown in (A) are representative analysis of the previous experiments (Fig. 4 in Tominaga and Kawamitsu, 2015*b*). Data are removed when *A* <±1.5 μmol m^−2^ s^−1^ in (A) and *A* <±0.4 μmol m^−2^ s^−1^ in (B) due to incorrect *D*_w/c_ (see Discussion). In (B), vertical and horizontal bars indicate ±SD (*n*=3).

To what extent does stomatal closure bring uncertainty to the calculation of *C*_i_? According to Eq. (6), we estimated *D*_w/c_ for the *A*–*C*_i_ curve measurements in sunflower leaves fed with ABA (Fig. 4 in Tominaga and Kawamitsu, 2015*b*) and plotted it against various values of ‘stomatal conductance’ (*g*_w_) (Fig. 8A). The *D*_w/c_ generally appeared to rise sharply as the *g*_w_ decreased from 100 mmol m^–2^ s^–1^. This trend is reasonable because cuticular conductance remains while stomatal conductance is reduced by stomatal closure. In addition, the *D*_w/c_ varied at a given *g*_w_ in different leaves as stomata closed (shown as different symbols in Fig. 8A), indicating variable cuticular conductance among leaves of a single plant (Kerstiens, 1996). This is further supported by the *g*_w_*–D*_w/c_ relationship for the adaxial side of common bean having the largest *D*_w/c_ despite the highest *g*_w_ (Fig. 8B) . The variation may be attributable to a lateral non-uniformity of cuticular conductance—the thinner cuticle of the guard cells likely has higher conductance than the cuticle on the surrounding epidermal cells (Šantrůček *et al.*, 2004). The cuticular conductance varied on the stomatous side (Fig. 8) perhaps with the properties of guard-cells/stomata (e.g. number, size, density), whereas it was relatively constant without stomata (Table 2). This possibility can be tested by analysing the *g*_w_*–D*_w/c_ relationships for leaves having various stomatal properties.

When using the regular assimilation chamber with gas flowing both adaxially and abaxially, *C*_i_ is calculated from the sum of fluxes on both sides. Therefore, hypostomatous or asymmetric amphistomatous leaves are not necessarily prone to the overestimation because the gas exchange occurring mostly on the stomatous side dilutes the gas exchange through the cuticle (as shown in the *A*–*C*_i_ measurements with a regular Li-Cor head set-up in Supplementary Fig. S5). However, the cuticular gas transport increases with the doubled cuticle. So, it is expected that the *D*_w/c_ illustrated in Fig. 8A departs from 1.6 more when gas-exchange measurements are configured normally.

In *A*–*C*_i_ curve measurements, ABA caused either no depression (Raschke and Hedrich, 1985) or moderate depression in the broad bean, *Vicia faba* (Terashima *et al.*, 1988). Likewise, *Xanthium strumarium* often showed significant depression (Fischer *et al.*, 1986; Daley *et al.*, 1989), but not always (Dubbe *et al.*, 1978; Mott and Takemoto, 1989). Contrasting results were also observed during experiments (Raschke and Hedrich, 1985; Mott, 1995). These reported experimental inconsistencies were not necessarily attributable to stomatal patchiness but perhaps to the extent of stomatal closure and/or variable cuticular conductance.

Stomatal patchiness may affect the calculation as lateral gas diffusion is obstructed in the intercellular airspace (Terashima *et al.*, 1988; Terashima, 1992). This is a trait of heterobaric leaves in which bundle-sheath extensions span the gap between the upper and the lower epidermis. Homobaric leaves lacking this barrier, on the other hand, have the interconnected airspace. All our measurements so far have been limited to heterobaric leaves. However, because of a similar arrangement, the cuticle does affect the calculation regardless of the internal anatomy.

### Considerations of the direct measurement

We technically altered the amphi- to hypostomatous leaves by attaching the cup on one side, thereby increasing the CO_2_ gradients (Parkhurst *et al.*, 1988; Boyer and Kawamitsu, 2011). The *C*_i(m)_ reflects an equilibrium with the concentration just beneath the interior of epidermis on the cup-attached side. On the other hand, the site for the *C*_i(c)_ may be peristomata beneath the chamber-attached side (Sharkey *et al.*, 1982; Mott and O’Leary, 1984; Parkhurst *et al.*, 1988), or it may be deeper inside the leaf (Nonami and Schulze, 1989; Brodribb *et al.*, 2007). In either case, *C*_i(m)_ represents the CO_2_ that has diffused through a somewhat longer path. This has been previously indicated by the *C*_i(m)_ being slightly but consistently lower than the *C*_i(c)_ in sunflower leaves with open stomata (Tominaga and Kawamitsu, 2015*a*,b; Boyer, 2015a,b). In contrast, the *C*_i(m)_ was almost identical to the *C*_i(c)_ in the present experiments (Figs 4 and 7A), suggesting little gradient. Because the gradients must be created by the mesophyll assimilation activity (i.e. *A*), this is likely due to slower assimilation under sub-saturating irradiance (300 μmol m^–2^ s^–1^). For the flipped leaves illuminated from the abaxial side, even slower mesophyll activity (indicated by the smaller *A*–*C*_i(m)_ slope) should have further contributed to the minor gradients (Fig. 7A).

The larger chamber has advantages over the smaller chamber owing to the shorter length (circumference) of the gasket relative to the enclosed leaf area. The leak effect should be reduced because gaseous pores between the rubber gaskets and leaf are the major path for leakage in commercial chambers (Flexas *et al.*, 2007). Diffusion leak could be further suppressed by using semi-solid materials for the gaskets. Consequently, our lab-made chamber enabled commercial open gas-exchange equipment to measure *in vivo* cuticle properties (Fig. 3). Nevertheless, the *g*_c_ derived from the *C*_i(m)_ became erratic as *C*_a_ decreased (Figs 5A and 6A). As *C*_a_ decreases, *g*_c_ becomes increasingly sensitive to the small *A*, for which the noise/signal ratio increases accordingly whereas the CO_2_ gradient (*C*_a_–*C*_i_) decreases [Eq. (5)]. On the other hand, the water vapor gradient (*w*_i_–*w*_a_) is relatively stable, and thus the calculation of *g*_w_ [Eq. (3)] is insensitive to change in *C*_a_ (see Supplementary Fig. S4). The signal noise in *A* also affects the estimation of *D*_w/c_ [Eq. (6)]. Thus, *D*_w/c_ is recommended to be estimated from multiple measurements when *g*_w_ is stable (Fig. 7B), or otherwise discarded in the region where *A* is small (as implemented in Fig. 8).

In clamp-on chambers, gas-exchange measurements may be affected by the edge—respiratory CO_2_, originating from the leaf areas below the gaskets, laterally diffuses through the intercellular airspace to the leaf areas inside the chamber (Jahnke and Krewitt, 2002; Pieruschka *et al.*, 2005). Because this internal CO_2_ supply tends to increase *C*_i_ around the edge while reducing the apparent *A* there (Pieruschka *et al.*, 2006), both measured flux and *C*_i(m)_ can be affected. The effect is detectable in homobaric leaves but not heterobaric leaves with negligible lateral diffusion (Pieruschka *et al.*, 2005, 2006). As with leakage through the gasket, the larger chamber/cup helps to avoid the intercellular leakage by decreasing the edge-to-area ratio of the enclosed leaf part.

### Implications for the photosynthesis model parameters

The impact of the cuticle carries over to the critical parameters in photosynthetic models (Hanson *et al.*, 2016). Because mesophyll conductance (*g*_m_) describes assimilatory CO_2_ drawdown from intercellular spaces to chloroplast (*C*_c_; i.e. *g*_m_=*A*/(*C*_i_–*C*_c_)), overestimation of *C*_i_ immediately leads to underestimation of *g*_m_. *C*_i_* and day respiration (*R*_d_) can also be affected by the overestimation of *C*_i_ as they are commonly estimated from the initial *A*–*C*_i_ slopes under multiple sub-saturating irradiances (Walker and Ort, 2015). Our Fig. 7A clearly shows the slope tilted by the overestimation .

Recently, a new method for rapid *A*–*C*_i_ response (RACiR) greatly reduced the measurement time of an *A*–*C*_i_ curve to ~5 min, and succeeded in estimating the same model parameters, namely the maximum rate of Rubisco carboxylation (*V*_c,max_) and electron transport (*J*_max_), as a standard *A*–*C*_i_ methodology (Stinziano *et al.*, 2017). The technique relies on technological advancements of a new instrument (LI-6800, Li-Cor) that allow high temporal resolution measurements under rapidly changing CO_2_ concentrations (Stinziano *et al.*, 2017). Whereas this rapid technique opens up the opportunity of high-throughput, large-scale, and on-site *A*–*C*_i_ measurements, the uncertainty of *C*_i_ still remains because the method similarly relies on the calculation. The direct measurement may be applicable to the rapid technique because the time required for the *C*_i(m)_ to reach equilibrium was as fast as the *C*_i(c)_ when CO_2_ was changed quickly (Figs 4B and 5B). However, the *C*_i(m)_ lagged behind the *C*_i(c)_ when the stomatal conductance for the cup-attached side would be low (Fig. 6B). For the RACiR featuring the direct measurement, the volume of the closed system ought to be reduced until the time lag is negligible.

### Conclusions and prospects

Overestimation of *C*_i_ is a general problem in leaf gas-exchange measurements. Obviously, there should be caution when stomatal closure is stimulated, e.g. under drought and salinity (Flexas *et al.*, 2004). Current techniques cannot distinguish cuticular gas transport from measured gas exchange on stomatous leaf surfaces. This makes the overestimation unpredictable. Assuming cuticle is a perfect barrier to CO_2_ (i.e. cuticular conductance to CO_2_ is zero), $g_{\text{w}}^{\text{cut}}$ can be estimated on the stomatous surface and used for correction (Boyer, 2015*b*; Tominaga and Kawamitsu, 2015*b*). However, the correction would be obstructed by the variability among leaves (Fig. 8). We conclude that direct measurements are a better option—especially when stomatal gas transport is restricted (Boyer, 2015*a**,b*; Tominaga and Kawamitsu, 2015*a*,b; Hanson *et al.*, 2016).

Given climate change will likely increase water scarcity worldwide, it is urgent to predict plant response to water deficit that primarily affects photosynthesis (Hanson *et al.*, 2016). To achieve this goal, it is essential to accurately estimate the photosynthetic model parameters in response to stressful environments. However, uncertainties in the calculation of *C*_i_ have affected interpretation of gas-exchange measurements (Flexas *et al.*, 2004), thereby preventing scientists from exploring a broad range of species and environmental conditions. The direct measurement may now be a solution. To play safe, *C*_i_ calculated from values of *g*_w_ <100 mmol m^−2^ s^−1^ should not be used (Fig. 8A). This range of *g*_w_ occurs in C_3_ leaves experiencing ‘severe drought’ that potentially imposes biochemical limitations (Medrano *et al.*, 2002; Flexas *et al.*, 2004). Recently, cuticular gas transport was found to be diminished by turgor loss due to a shrinkage effect (Boyer, 2015*a*). This may favor a minor cuticle effect on the calculation for dehydrated leaves. In this regard, the drought response of the cuticle needs to be investigated especially in intact leaves.

Now that improvement of gas exchange analysis largely depends on the capability of commercial instruments (Stinziano *et al.*, 2017), the built-in system is advantageous (Tominaga and Kawamitsu, 2015*a*). In addition, it is available to many scientists. The challenge is to match the system with emerging techniques.

## Supplementary data

Supplementary data are available at *JXB* online.

Fig. S1. Measurement of the volume of the closed system.

Fig. S2. Leak test in the closed system.

Fig. S3. Traces of temperature responses of cuticular transpiration and *C*_i_.

Fig. S4. Comparisons of *g*_c_ with *g*_w_.

Fig. S5. Plotting of *A*–*C*_i_ curves with our previous system.

Supplementary Figures
