# Supplementary material for: Direct measurement of intercellular CO2 concentration in a gas-exchange system resolves overestimation using the standard method
Source: J Exp Bot. 2018 Feb 8;69(8):1981–91. doi: 10.1093/jxb/ery044 (PMC6018834; doi:10.1093/jxb/ery044)
Supplement: Supplementary Figures [file ery044_suppl_supplementary_figures.pdf]

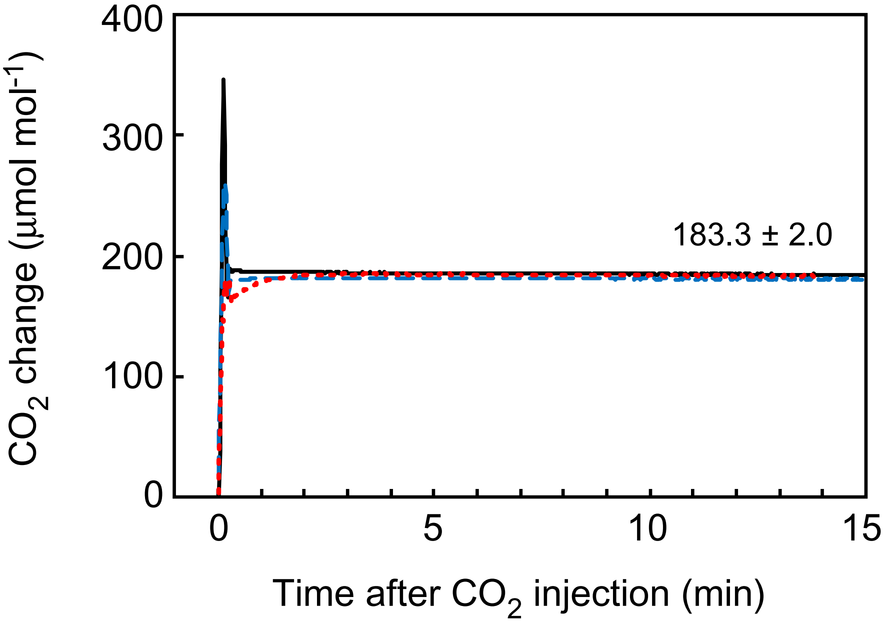

Fig. S1. Change of CO_2 in the closed loop after 0.5 ml of 5% CO_2 was injected through the water seal of the condenser (dew capture) in the closed loop. The injection was done with aluminum foil clamped instead of the leaf, and the seal was ensured with a coating of paraffin/lanolin. The experiment was replicated three times with different injection speeds. Solid, broken, and dotted lines indicate the slow, moderate, and rapid injections, respectively. Regardless of the injection speed, CO_2 increased essentially to the same degree with a mean of 183 ± 2.0 µmol mol^{-1}. From the data presented in this figure, the volume of the closed loop was estimated to be 137.2 ± 2.0 ml, whereas the volume for the previous system with a small cup was 117 ml (estimated from Fig. 3 in Tominaga and Kawamitsu, 2015*a*). It was apparent that the pressure caused by the injection slightly pushed down the water seal, but the volume of the injection was too small to push out a bubble. Thus, the injection increased the pressure somewhat inside the cup. Because the injection volume was <1% of the total volume, the pressure effect on the CO_2 readings was considered negligible.

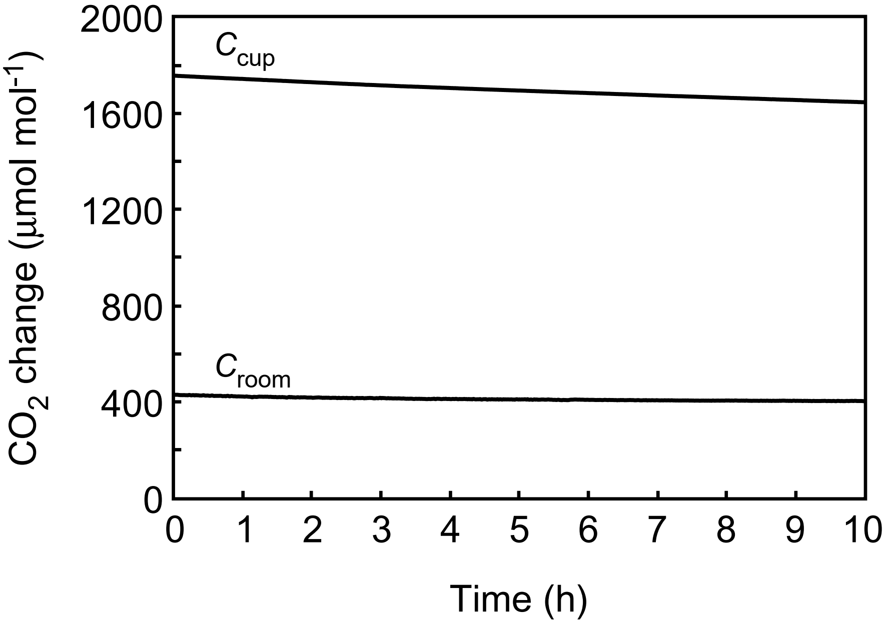

Fig. S2. Change in CO_2 in the closed loop (C_{cup}) after partially enclosing expired air containing high CO_2. The cup was closed with aluminum foil clamped instead of the leaf, and the seal was ensured with a coating of paraffin/lanolin. The C_{cup} decreased by 108 µmol mol^{-1} over 10 h while the CO_2 concentration outside the chamber (C_{room}) remained constant at ~400 µmol mol^{-1}. With the estimated volume of 137 ml in the closed system (Fig. S1), the apparent CO_2 flux (assimilation rate) was estimated to be 0.0054 µmol m^{-2} s^{-1}.

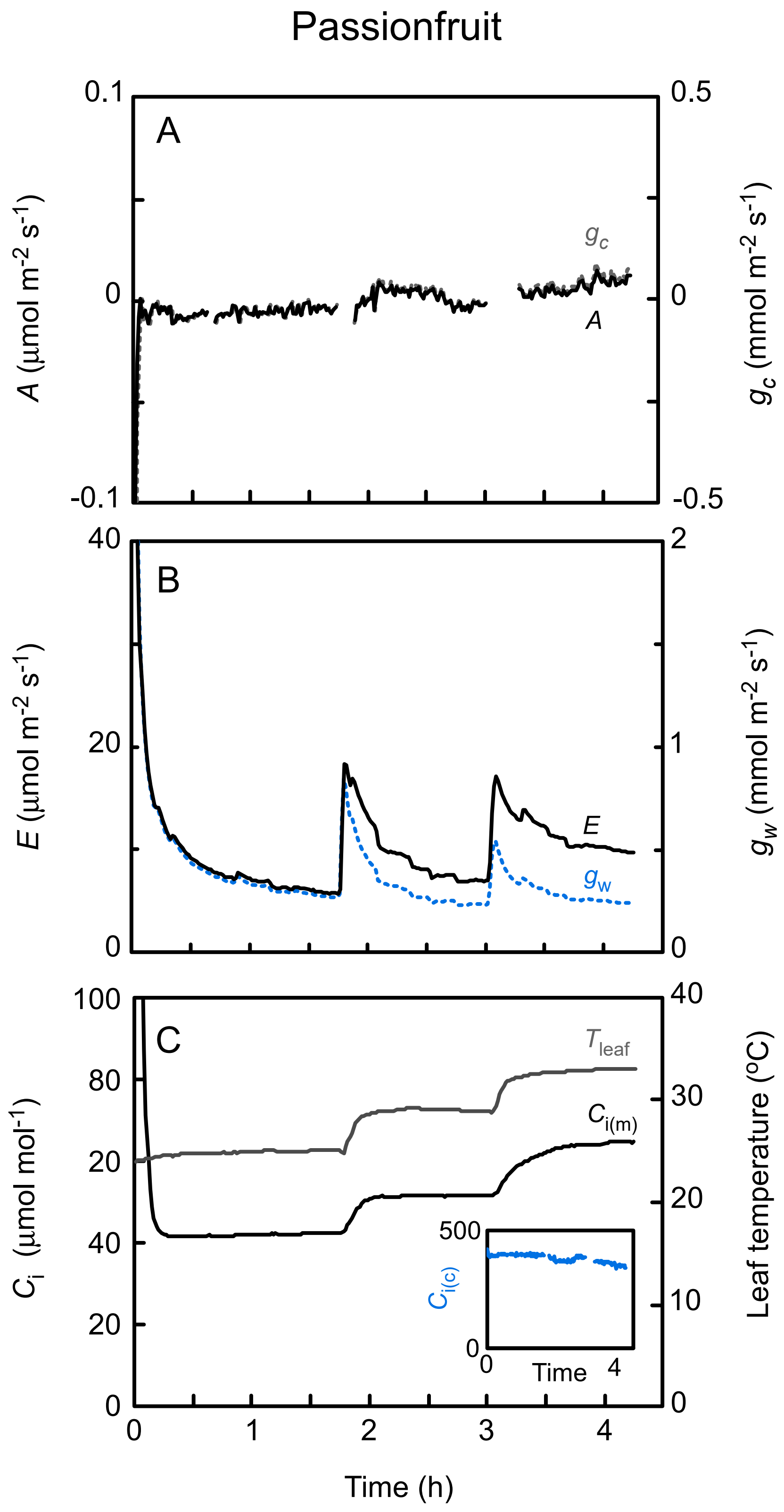

Fig. S3. Change in (A) assimilation rate (*A*) and conductance of CO_2 (g_c), (B) transpiration rate (*E*) and conductance of water vapor (g_W), and (C) intercellular CO_2 concentration (C_i) for a clamped passionfruit leaf. Leaf temperature was increased stepwise as shown in (C). Inset of (C) shows the change in $C_{i(c)}$ in the same experiment. Note that, at steady state, *E* increased with increasing leaf temperature while g_W was relatively constant. Representative experiment from four replications.

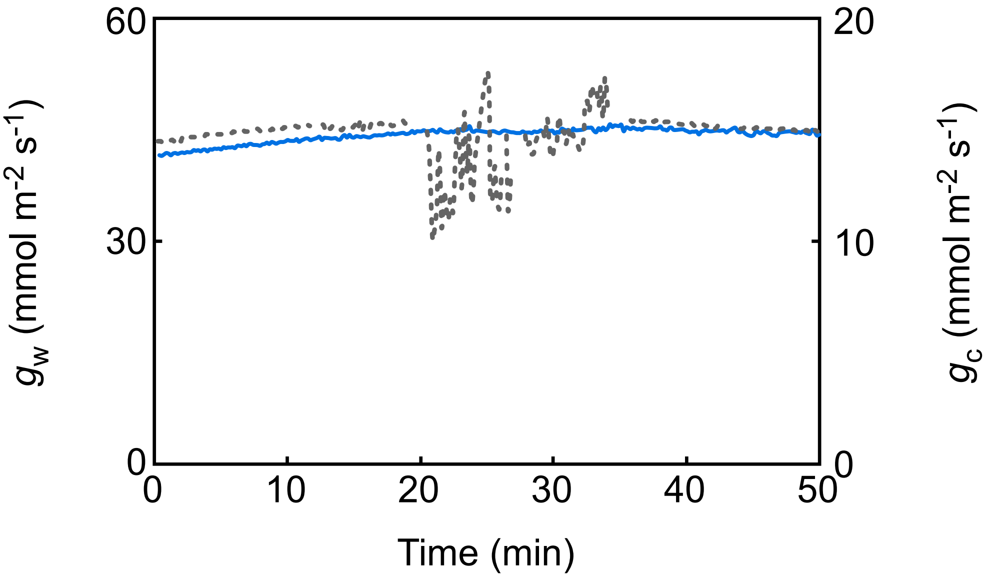

Fig. S4. Comparisons of the conductance of CO_2 (g_c) with that of water vapor (g_w) in Fig. 5A. The y axes were adjusted for one another.

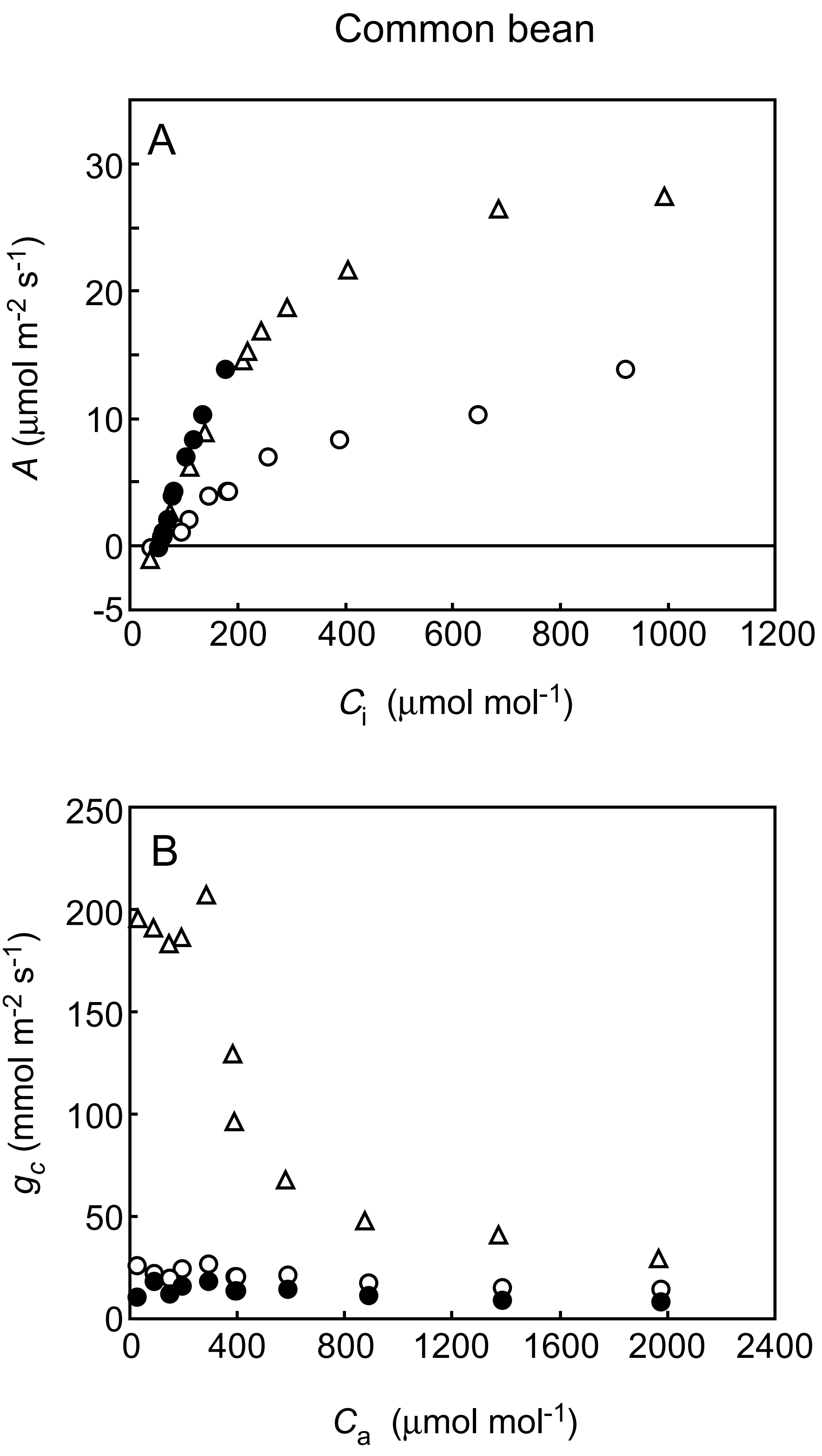

Fig. S5. (A) Comparison of A-C_i curves for common bean leaves measured with the LI-6400-40 chamber (open triangles) or the same chamber with the lower chamber having been replaced with the cup (open circle), and $C_{i(c)}$ was calculated from both sides or only the adaxial side of the leaf, respectively. Included in the comparison are $C_{i(m)}$ with the attached cup (closed circles). (B) Conductance of CO_2 (g_c) at various concentrations of ambient CO_2 (C_a) for the leaf in (A). Data were obtained in a series of measurements using a single leaf.
